# A CD68-expressing tissue-homing subset in peripheral CD8^+^ T cells predicts response to anti-PD-1 therapy in lung cancer

**DOI:** 10.3389/fimmu.2026.1995008

**Published:** 2026-10-13

**Authors:** Ying Wang, Jing Huang, Zhengyi Sun, Yumei Pei, Jiancheng Xu

**Affiliations:** Department of Laboratory Medicine, Infectious Diseases and Pathogen Biology Center, The First Hospital of Jilin University, Changchun, Jilin, China

**Keywords:** anti-PD-1 immunotherapy, CD68, CD8^+^ T cells, lung cancer, lysosome

## Abstract

**Background:**

T-cell function is regulated by immunometabolism, and lysosomes are signaling hubs. CD68 (LAMP4), traditionally regarded as a monocyte/macrophage marker, has been largely neglected in T cell biology. We aimed to assess the link between CD68 expression in T cells and anti-PD-1 response in lung cancer, and its potential as an early dynamic biomarker in peripheral blood CD8^+^ T cells.

**Methods:**

We analyzed transcriptomic (GSE126044, GSE135222, GSE166449) and single-cell (GSE207422) data from anti-PD-1-treated lung cancer cohorts to identify lysosome related pathways and key genes, validated with peripheral blood CD8^+^ T cell data (GSE111414). Prospectively, we enrolled lung cancer patients receiving anti-PD-1 therapy, collecting peripheral blood at baseline and week 3. Flow cytometry measured CD68 in CD8^+^ T cells and subsets, assessing association with response, progression-free survival (PFS), and functional markers (granzyme B, perforin, Ki-67, CD69, IFN-γ, IL-1β).

**Results:**

Responders showed enrichment of lysosome pathways and upregulation of 8 lysosome related genes including CD68, positively correlated with monocyte and T cell infiltration. Single-cell data linked CD68 expression in T cell to favorable response. After one cycle, the proportion of CD8^+^CD68^+^ T cells was lower in non-responders (*P<0.05*), with AUC 0.803 (*P=0.044*) and cutoff 1.015%, stratifying PFS (*P=0.0225*). CD68 was highest in naïve T cells at baseline, but decreased across subsets after treatment in non-responders (all *P<0.01*). Functionally, compared with CD8^+^CD68^-^ T cells, CD8^+^CD68^+^ T cells exhibited lower expression of granzyme B and perforin but higher expression of Ki-67, CD69, IFN-γ, and IL-1β (all *P<0.001*), suggesting a CD8^+^ T cell state characterized by lower cytotoxicity and enhanced proliferative and cytokine producing capacities, accompanied by a tissue residency associated phenotype.

**Conclusion:**

CD8^+^CD68^+^ T cells exhibit lower cytotoxicity but greater proliferative and cytokine producing capacities, together with a tissue residency associated phenotype, suggesting a precursor-like state. Low CD68 expression in tumor-infiltrating T cells and the post-treatment reduction of CD68 expression in peripheral blood CD8^+^ T cells are associated with resistance to anti-PD-1 therapy in lung cancer. These findings suggest that CD8^+^CD68^+^ T cells may serve as a potential early dynamic immune biomarker for monitoring and assessing response to anti-PD-1 therapy in lung cancer.

## Introduction

Lung cancer remains the leading cause of cancer related morbidity and mortality worldwide. Although immune checkpoint inhibitors, particularly anti-PD-1 antibodies, have achieved substantial clinical benefits in a subset of patients with lung cancer, resistance remains a major challenge limiting therapeutic efficacy (1, 2). T lymphocytes are central effectors of adaptive antitumor immunity, and their functional states, including activation, proliferation, differentiation, and tissue homing, are closely associated with responses to immunotherapy (3, 4). Increasing evidence indicates that T cell function and differentiation are continuously shaped by intracellular metabolic states, with metabolic reprogramming playing a critical role in maintaining T cell function and enabling adaptation to the tumor microenvironment (3, 4).

The lysosome is not only a major organelle responsible for the degradation of intracellular components but also an important platform for nutrient sensing, metabolic regulation, and signal integration, thereby contributing to the maintenance and functional reprogramming of immune cells (5). In T cells, lysosome associated processes are involved in intracellular degradation, autophagy, membrane protein trafficking, and metabolic signaling, and may consequently influence T cell activation, proliferation, and effector functions. Many of these lysosome dependent processes are mediated by lysosome associated membrane proteins (LAMPs). Members of the LAMP family, including LAMP1, LAMP2, and LAMP4/CD68, are transmembrane proteins predominantly localized to lysosomal and endosomal membranes and have been implicated in the regulation of immune cell functions (6, 7). Notably, CD68 (also known as LAMP4) has traditionally been regarded as a canonical marker of the monocyte/macrophage lineage (8, 9). However, publicly available resources, including the Human Protein Atlas, indicate that CD68 expression can also be detected in T cells, suggesting that CD68 may have a role in T cell function. Nevertheless, its expression patterns and potential biological significance across distinct T cell subsets remain largely unexplored.

In our preliminary analyses of multi-cohort transcriptomic and single-cell RNA sequencing datasets, we found that multiple lysosome related pathways were significantly enriched in the tumor microenvironment of patients who responded to anti-PD-1 therapy. We further observed an association between CD68 expression in T cells and clinical benefit from anti-PD-1 treatment. In peripheral blood, CD68 expression in CD8^+^ T cells was positively associated with clinical treatment response and progression-free survival (PFS) following anti-PD-1 therapy. Given that peripheral blood sampling is minimally invasive, readily accessible, and amenable to longitudinal monitoring during treatment, the present study focuses on CD68 expression in peripheral blood CD8^+^ T cells. We systematically characterize CD68 expression across distinct T cell differentiation subsets and investigate its associations with molecular features related to T cell activation, proliferation, and tissue residency. Furthermore, we preliminarily evaluate the potential of CD68 expression in peripheral blood CD8^+^ T cells as a candidate predictive biomarker of response to anti-PD-1 therapy. Collectively, this study aims to provide a basis for further elucidating the relationship between lysosome associated T cell states and responses to immunotherapy in lung cancer.

## Methods

### Transcriptomic profiling of the tumor microenvironment

Three lung cancer anti–PD-1 immunotherapy datasets (GSE126044, GSE135222 and GSE166449) were downloaded from the GEO database. Multi-batch integration and normalization were performed with the R packages limma, edgeR and sva, yielding a merged cohort of 20 responders and 45 non-responders that was subsequently used for tumor microenvironment (TME) transcriptome analyses. Dataset GSE207422 served as an independent validation set for transcriptome profiling of the tumor microenvironment in anti–PD-1 treated lung cancer patients.

Ten lysosome related immune associated pathways, including Tuberculosis (map05152), Phagocytosis (map04145), Antigen processing and presentation (map04612), Glycosaminoglycan degradation (map00531), Lysosome biogenesis (map04142), Toll-like receptor signaling pathway (map04620), Apoptosis (map04210), Autophagy-animal (map04140), Endocytosis (map04144), and mTOR signaling pathway (map04150), along with their constituent genes, were retrieved from the Molecular Signatures Database v7.4 (https://www.gsea-msigdb.org/gsea/msigdb). Gene set enrichment analysis (GSEA) of these pathways in the tumor microenvironment was performed using the clusterProfiler package. Subsequently, differential gene expression within the map04142_Lysosome biogenesis pathway was analyzed with the limma package. Associations between therapeutic efficacy and immune cell infiltration levels were evaluated using microenvironment cell populations counter. Finally, Spearman rank correlations between eight prominent lysosomal genes and immune cell infiltration scores were calculated and visualized with the igraph and ggplot2 packages.

### Single-cell transcriptomic analysis of intratumoral T cells

The single-cell transcriptomic data were obtained from the GEO database under accession number GSE207422. All data processing and analysis were performed in the R environment using the Seurat package. During the quality control phase, we applied initial filtering criteria requiring genes to be detected in at least three cells. Cells were further excluded if they exhibited the following characteristics: mitochondrial gene content exceeding 20%, ribosomal gene content above 50%, or a total detected gene count either below 200 or above 6,000. This stringent filtering ensured that subsequent analyses were based on high quality cells. In the preprocessing stage, gene expression values were normalized using the “LogNormalize” method. The top 2,000 highly variable genes were then identified through the “vst” method in the “FindVariableFeatures” function. These genes were subsequently standardized using the “ScaleData” function to remove technical batch effects, followed by principal component analysis for dimensionality reduction based on the standardized highly variable genes.

To avoid bias from baseline group mismatches, pre-treatment biopsy samples were excluded from this study. This decision was made because pre-treatment samples were only available from subsequent treatment responders (R group), while corresponding samples from non-responders (NR group) were lacking, preventing valid paired comparisons. Therefore, all analyses were conducted using post-treatment samples, ultimately including 4 responders and 8 non-responders. T cell populations were identified based on CD3D and CD3E expression characteristics, with particular focus on analyzing the expression patterns of seven lysosome related genes, including CD68, tartrate-resistant acid phosphatase 5 (ACP5), cathepsin S (CTSS), cathepsin L (CTSL), cathepsin W (CTSW), lysosomal protein transmembrane 5 (LAPTM5), and solute carrier family 11 member 1 (SLC11A1). For statistical analysis, independent samples t tests were employed to compare gene expression differences between responder and non-responder groups. Spearman correlation analysis was used to examine the relationship between CD68 expression levels in T cells and treatment efficacy, with response status treated as an ordinal variable. Multiple comparisons were adjusted using the Benjamini-Hochberg method, with statistical significance defined as an adjusted p value < 0.05.

### Transcriptomic analysis of peripheral CD8^+^ T cells

To explore the relationship between peripheral immune status and clinical outcomes, we retrieved the GSE111414 dataset, which comprises RNA-seq data from peripheral CD8^+^ T cells obtained from five responders and five non-responders at baseline and after one anti-PD-1 cycle. Within each clinical subgroup, differential expression analysis between baseline and post-treatment samples was performed using DESeq2. Genes retaining ≥10 counts in the smallest subgroup were kept, and the false discovery rate was applied for multiple testing correction to ensure robustness. CD68 expression levels across the comparison groups were plotted using GraphPad Prism 10.

### Patient recruitment

This study recruited patients with pathologically confirmed advanced (stage III/IV) lung cancer who were treated at the First Hospital of Jilin University (Changchun) from November 2024 to April 2025. All participants received intravenous PD-1 monoclonal antibody every three weeks, and treatment was continued until radiographically documented disease progression or the onset of unacceptable toxicity. According to RECIST v1.1, patients were classified as responders (R) or non-responders (NR). R included those who achieved a complete response (CR), partial response (PR), or experienced stable disease (SD) lasting at least 24 weeks during anti-PD-1 therapy. NR were defined as those who progressed within the first 24 weeks of treatment. Progression-free survival (PFS) was calculated from the date of initial medication to the first occurrence of disease progression or death of any cause. The study protocol was approved by the Human Ethics Committee of the First Hospital of Jilin University (Approval No. 24k285-001), and all participants provided written informed consent before sample collection.

### Sample collection

Peripheral blood specimens were obtained both before the initial anti-PD-1 infusion and after the first cycle (week 3). Peripheral blood mononuclear cells (PBMCs) were subsequently isolated via density gradient centrifugation and immediately subjected to flow cytometric analysis.

### Flow cytometry analysis

We analyzed PBMCs using a FACSCanto™ II flow cytometer to measure the expression of multiple biomarkers. These included surface markers such as CD3, CD8, CD45RA, CCR7, and CD69, along with intracellular proteins like CD68, perforin, granzyme B, IFN-γ, and IL-1β, and the nuclear antigen Ki-67. To rigorously exclude contamination from monocytes, macrophages, and other non T lineages, a strict sequential gating strategy was applied in all analyses: lymphocytes were first gated by FSC-A vs. SSC-A; subsequently, the CD8^+^ T cell population was strictly defined as CD3^+^CD8^+^ double positive cells, and only this population was used for downstream quantification of CD68 and all other intracellular markers. Flow cytometric data were collected and analyzed using BD FACSDiva™ software, and the corresponding gating strategies are illustrated in the figures. All antibodies applied in this study were purchased from well-known commercial suppliers, namely BD Biosciences, R&D Systems, and Santa Cruz Biotechnology. For the detection of intracellular cytokines, PBMCs were first incubated with BD Leukocyte Activation Cocktail (supplemented with BD GolgiPlug) at 37 °C for 4 hours. After this stimulation, the cells were fixed and permeabilized using the Transcription Factor Buffer Set, and then subjected to intracellular staining according to the manufacturer’s recommendations. Note that this assay measures total intracellular CD68 protein and cannot distinguish lysosome-membrane-bound versus soluble CD68 isoforms.

### Statistical analysis

Normality was assessed using the Shapiro-Wilk test. For two-group comparisons, Student’s t test or Mann-Whitney U test was used for unpaired data, and paired t test or Wilcoxon signed-rank test for paired data, as appropriate. For multiple-group comparisons, one-way ANOVA or Kruskal-Wallis test was used for independent data, and Friedman test was used for paired data. Two-way ANOVA was used for comparisons involving time and response status. We defined statistical significance as *P < 0.05, **P < 0.01, and ***P < 0.001. We also used receiver operating characteristic (ROC) curves to test whether the proportion of circulating CD8^+^CD68^+^ T cells could serve as a predictor of the resistance to treatment. The optimal threshold drawn from the ROC analysis was then utilized to dichotomize patients into high risk and low risk subsets for anti-PD-1 resistance. Kaplan-Meier plots were generated to display PFS distributions between the two risk strata, and intergroup comparisons were performed using the log-rank test. All statistical procedures were implemented in GraphPad Prism 10.0 (RRID: SCR_002798) and SPSS 27.0 (RRID: SCR_002865).

## Results

### Correlation between CD68 associated lysosomal signaling pathways in tumors and efficacy of anti-PD-1 immunotherapy in lung cancer

To investigate the role of lysosome related pathways in anti-PD-1 therapy for lung cancer, we integrated transcriptomic data from tumor samples across three independent cohorts (GSE126044, GSE135222, and GSE166449), comprising a total of 65 patients, including 20 responders (R) and 45 non-responders (NR), and performed differential expression analysis. Batch correction was performed before the analysis, and the principal component analysis (PCA) score plots before and after correction are shown in **Supplementary Figure S1**. Gene set enrichment analysis (GSEA) revealed significant enrichment of multiple lysosome related pathways in the R group, including Tuberculosis (map05152), Phagocytosis (map04145), Antigen processing and presentation (map04612), Glycosaminoglycan degradation (map00531), Lysosome biogenesis (map04142), Toll-like receptor signaling pathway (map04620), Apoptosis (map04210), Autophagy-animal (map04140), Endocytosis (map04144), and mTOR signaling pathway (map04150) (**Figure 1A**). We next focused on the 120 genes included in the map04142_Lysosome biogenesis pathway (**Supplementary Table S1**). Notably, canonical lysosomal markers such as LAMP1 and LAMP2 did not exhibit significant differential expression between the R and NR groups in our integrated analysis. Instead, volcano plot analysis identified eight genes that were significantly upregulated in the R group, namely LAPTM5, CD68, CTSS, CTSW, ACP5, CTSL, SLC11A1, and ATPase H+ Transporting V0 Subunit D2 (ATP6V0D2) (**Figure 1B**).Heatmap analysis further showed that six of these genes (LAPTM5, CD68, CTSS, CTSW, ACP5, and CTSL) were significantly positively correlated with the inferred abundance of monocytes and T cells based on RNA-seq data (R > 0.5; **Figure 1C**). These findings were validated in an independent cohort (GSE207422), where the map04142_Lysosome biogenesis pathway activity remained elevated, and seven genes (all except CTSL) maintained positive correlations with monocytic and T cell abundance (**Supplementary Figure S2**).

**Figure 1:**
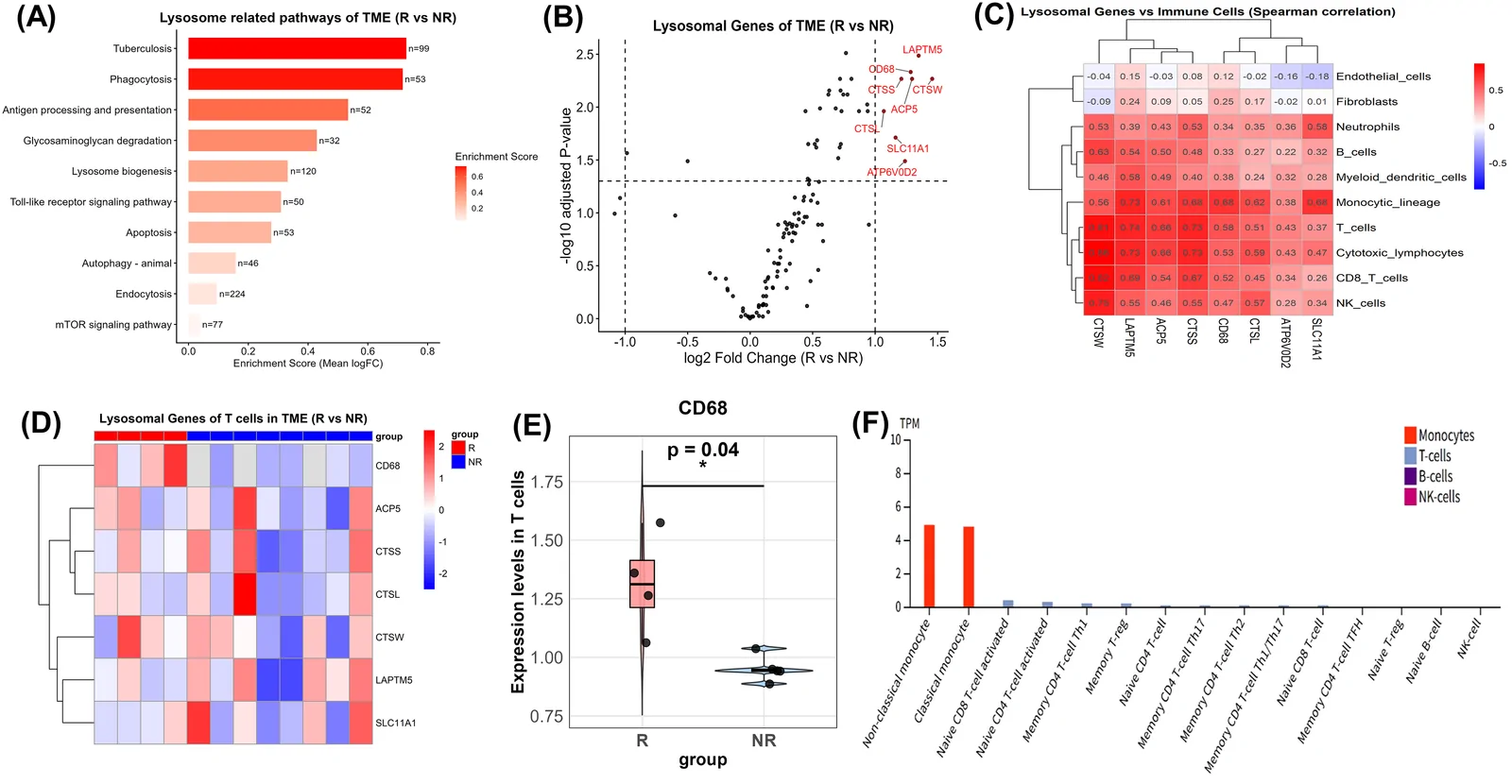
Analysis of the lysosomal pathway and associated genes in an antI-PD-1 immunotherapy cohort for lung cancer. **(A)** Enrichment analysis of lysosome-related pathways in the responder (R) versus non-responder (NR) groups from the merged cohort (GSE126044, GSE135222, GSE166449). **(B)** Volcano plot depicting differentially expressed genes of the map04142_Lysosome biogenesis pathway between the two groups. Thresholds were set at *P < 0.05* and |log2FC| > 1, with upregulated genes highlighted in red. **(C)** Heatmap showing the correlation analysis between eight significantly upregulated lysosomal genes and the infiltration levels of 10 immune cell types in the tumor microenvironment. **(D)** Heatmap illustrating the correlation between the expression of the eight lysosomal genes in T cell subsets, analyzed based on single-cell RNA sequencing data, and therapeutic response. **(E)** Comparison of CD68 gene expression levels in T cells between R and NR groups. Mann-Whitney U test, *P < *0.05*. **(F)** Expression levels of CD68 protein in different immune cell types according to the Human Protein Atlas database.

To determine whether the upregulation of these lysosome related genes occurred in T cells, we further analyzed single-cell RNA sequencing data from the GSE207422 cohort. Among the lysosome related genes examined, only CD68 expression in T cells was significantly associated with a favorable treatment response (**Figure 1D**). Consistently, tumor-infiltrating T cells from responders (n=4) exhibited significantly higher CD68 expression than those from non-responders (n = 8; *p < 0.04*; **Figure 1E**). Furthermore, single-cell data from the Human Protein Atlas revealed that CD68 protein levels were markedly higher in activated naïve CD4^+^ and CD8^+^ T cells than in naïve T cells (**Figure 1F**). Collectively, these findings from publicly available multi-cohort transcriptomic and single-cell sequencing datasets consistently demonstrate that CD68 expression in tumor-infiltrating T cells is positively associated with response to anti-PD-1 therapy in patients with lung cancer, suggesting that CD68 may be related to treatment associated changes in T cell functional states.

### Reduced CD68 expression in peripheral CD8^+^ T cells after anti-PD-1 therapy predicts non-response and shorter PFS

Given the association between CD68 expression in tumor-infiltrating T cells and treatment efficacy, we further investigated whether dynamic changes in CD68 expression in peripheral blood CD8^+^ T cells were similarly associated with clinical response. To this end, we analyzed the GSE111414 dataset, which included peripheral blood CD8^+^ T cell samples from five responders and five non-responders collected at baseline and after the first treatment cycle. After completion of the first treatment cycle, CD68 expression in CD8^+^ T cells was significantly lower in non-responders than in responders (*P < 0.05*; **Figure 2A**). To validate this finding, we enrolled an independent cohort of 17 patients with lung cancer and longitudinally quantified CD68 expression in peripheral blood CD8^+^ T cells. Baseline patient characteristics are summarized in **Table 1**. Peripheral blood samples were collected at baseline and at the end of the first treatment cycle (week 3), followed by PBMC isolation and flow cytometric analysis. Notably, CD68 was assessed using intracellular staining after fixation and permeabilization to quantify total CD68 protein levels in CD8^+^ T cells (**Figure 2B**). The proportion of CD8^+^CD68^+^ T cells (CD8 as a surface marker and CD68 as an intracellular marker) remained relatively stable in responders before and after treatment, whereas this proportion decreased significantly after treatment in non-responders (*P < 0.05*; **Figure 2C**). We next investigated whether the post-treatment proportion of CD8^+^CD68^+^ T cells could serve as a potential predictor of resistance to anti-PD-1 immunotherapy. Receiver operating characteristic (ROC) curve analysis yielded an area under the curve (AUC) of 0.8030 (*P = 0.0444*; **Figure 2D**), indicating moderate discriminatory performance. The optimal cutoff value was 1.015%, corresponding to a sensitivity of 66.67% and a specificity of 90.91% for identifying treatment resistance. Based on this cutoff, patients were stratified into high and low risk groups, and PFS was subsequently compared between the two groups. Kaplan-Meier analysis revealed a statistically significant difference in PFS between the two groups (*P = 0.0225*; **Figure 2E**). Collectively, these findings demonstrate that a decrease in the proportion of peripheral blood CD8^+^CD68^+^ T cells after one cycle of anti-PD-1 therapy is associated with non-response and shorter PFS. These results further suggest that early dynamic changes in CD68 expression in peripheral blood CD8^+^ T cells may have potential value in reflecting the efficacy of anti-PD-1 therapy in patients with lung cancer.

**Figure 2:**
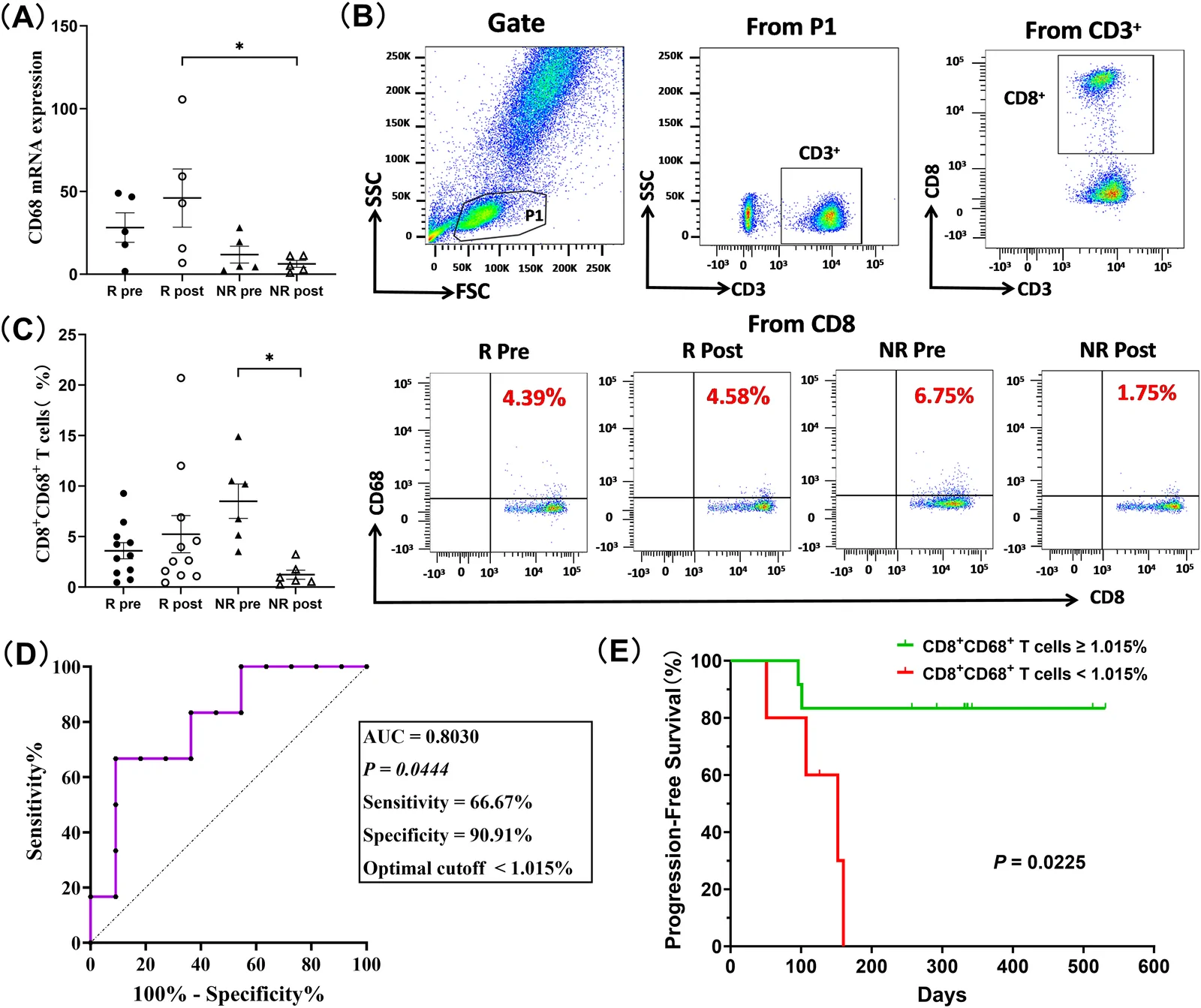
Loss of CD68 in peripheral CD8^+^ T cells predicts poor response to antI-PD-1 therapy. **(A)** Analysis of CD68 mRNA expression levels in peripheral CD8^+^ T cells based on the GSE111414 dataset. Samples were divided into four groups (n=5 per group): pre-treatment samples from responders (R Pre), post-treatment samples from responders (R Post), pre-treatment samples from non-responders (NR Pre), and post-treatment samples from non-responders (NR Post). Two-way ANOVA, *P < *0.05*. **(B)** Schematic of the flow cytometry analysis strategy. Top: Gating strategy for identifying the CD8^+^CD68^+^ T cell subset from PBMCs; Bottom: Representative flow cytometry plots from R and NR before and after treatment. **(C)** Quantitative comparison of the frequency of peripheral blood CD8^+^CD68^+^ cells in R (n=11) versus NR (n=6) before and after treatment. Two-way ANOVA, *P < *0.05*. **(D)** ROC curve evaluating the predictive value of post-treatment CD8^+^CD68^+^ T cell proportion for resistance to anti-PD-1 immunotherapy (AUC = 0.8030, *P = 0.0444*). **(E)** Kaplan-Meier analysis of progression-free survival in patients stratified by post-treatment CD8^+^CD68^+^ T cell proportion using the 1.015% cutoff.

**Table 1:** Clinicopathologic characteristics of anti-PD-1 treated lung cancer patients.

Patient characteristics	All	Responders	Non-responders
**Number**	17	11	6
**Age: Median(Range)**	58(46~81)	57(46~81)	60.5(48~67)
Sex, No. (%)			
Male	12	7	5
Female	5	4	1
Disease stage, No. (%)			
c-stage III	5	3	2
c-stage IV	12	8	4
Response, No. (%)			
CR	0	0	0
PR	10	10	0
SD	1	1	0
PD	6	0	6

### Widespread downregulation of CD68 across CD8^+^ T cell differentiation subsets in non-responders

To determine which CD8^+^ T cell subsets express CD68, we first classified CD8^+^ T cells into four major differentiation subsets by flow cytometry based on the surface expression of CCR7 and CD45RA (**Figure 3A**): naïve T cells (TN, CD45RA^+^CCR7^+^), central memory T cells (TCM, CD45RA^−^CCR7^+^), effector memory T cells (TEM, CD45RA^−^CCR7^−^), and terminally differentiated effector memory T cells re-expressing CD45RA (TEMRA, CD45RA^+^CCR7^−^). We then analyzed baseline samples from 13 patients receiving anti-PD-1 therapy to characterize the distribution of CD68 expression across these CD8^+^ T cell subsets. At baseline, CD68 expression was highest in TN cells and was significantly higher than that in TEM (*P < 0.01*) and TEMRA (*P < 0.05*) subsets (**Figure 3B**). Following the first treatment cycle, this expression pattern was altered. CD68 expression in TN cells decreased, and the previously observed differences between TN cells and the other CD8^+^ T cell subsets were no longer evident (**Figure 3C**). Notably, after anti-PD-1 therapy, CD68 expression was significantly downregulated in all four CD8^+^ T cell subsets, including TN, TCM, TEM, and TEMRA cells, in patients who failed to respond to treatment (all *P < 0.01*; **Figures 3D–G**). Collectively, these findings demonstrate that the post-treatment decrease in CD68 expression is not restricted to a specific CD8^+^ T cell differentiation subset but occurs broadly across distinct differentiation states in non-responders. This widespread downregulation suggests that dynamic changes in CD68 may be associated with coordinated remodeling of multiple CD8^+^ T cell states during anti-PD-1 therapy.

**Figure 3:**
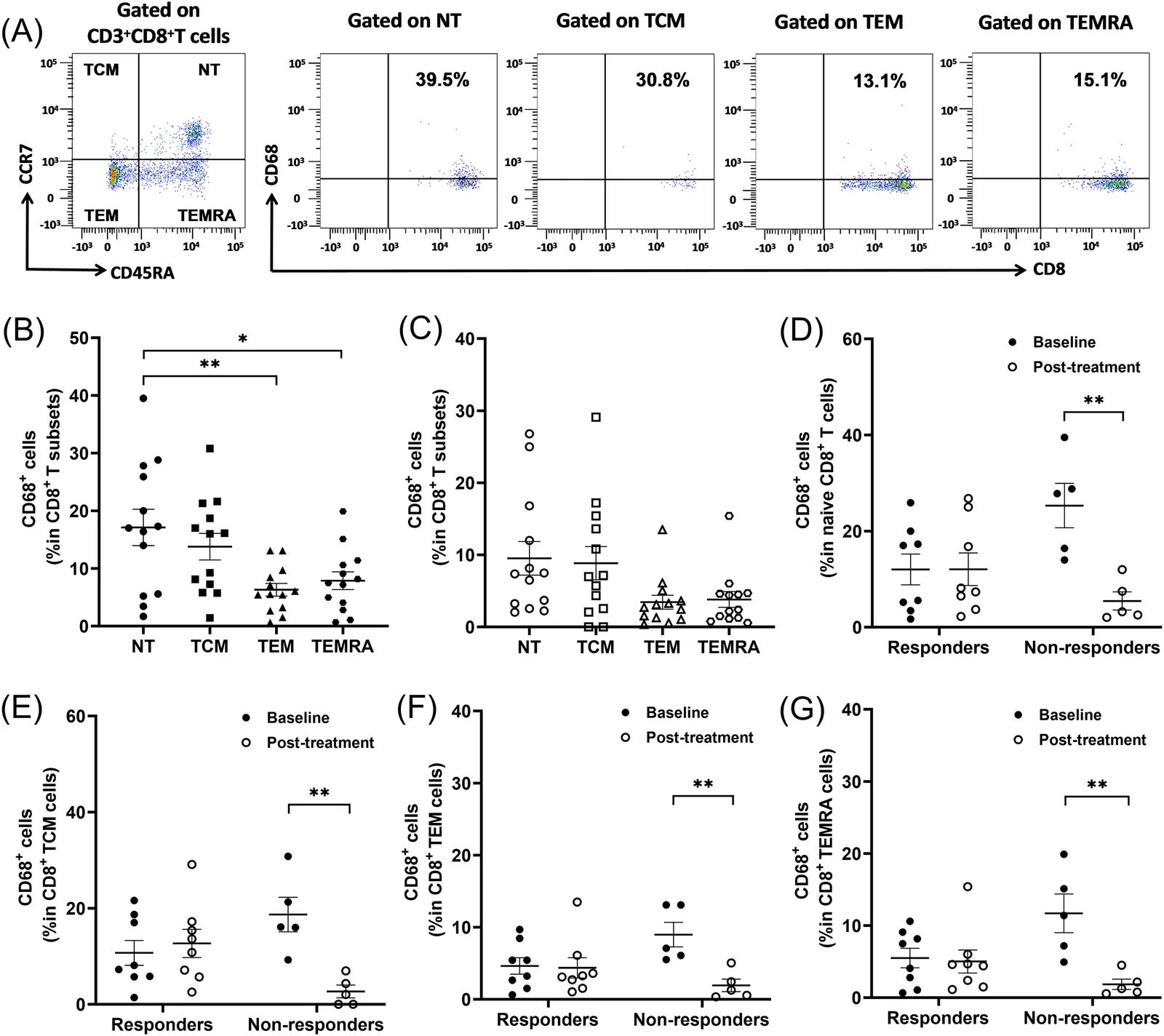
CD68 expression dynamics across CD8^+^ T cell subsets during anti-PD-1 therapy. **(A)** Gating strategy for identifying CD8^+^ T cell subsets based on CCR7 and CD45RA expression, and subsequent analysis of CD68 expression within each subset: naïve T cells (TN), central memory T cells (TCM), effector memory T cells (TEM), and terminal effector memory re-expressing CD45RA (TEMRA). **(B)** Baseline CD68 expression levels across CD8^+^ T cell subsets in pre-treatment samples (n=13). One-way ANOVA, *P < 0.05, **P < 0.01. **(C)** CD68 expression in each subset after one cycle of anti-PD-1 treatment. **(D-G)** Comparison of CD68 expression in **(D)** TN, **(E)** TCM, **(F)** TEM, and **(G)** TEMRA subsets between responders (n=8) and non-responders (n=5) at baseline and post-treatment. Two-way ANOVA, *P < *0.05*, **P < *0.01*.

### Enhanced proliferative and tissue resident functional features of peripheral blood CD8^+^CD68^+^ T cells

To investigate the relationship between CD68 expression and the functional state of CD8^+^ T cells, we stratified peripheral blood CD8^+^ T cells according to CD68 expression and compared the expression of molecules associated with cytotoxicity, proliferation, tissue residency, and effector functions. PBMCs collected from 20 patients with lung cancer after completion of the first cycle of anti-PD-1 therapy were analyzed, with the gating strategy shown in **Figure 4A**. Compared with CD8^+^CD68^−^ T cells, CD8^+^CD68^+^ T cells exhibited significantly lower expression of the cytotoxic molecules granzyme B and perforin (**Figures 4B, C**; *P < 0.001* for both). In contrast, CD8^+^CD68^+^ T cells displayed enhanced proliferative and tissue residency associated phenotypes, as evidenced by significantly higher expression of the proliferation marker Ki67 and the tissue residency associated molecule CD69 (*P < 0.001* for both; **Figures 4D, E**). In addition, CD8^+^CD68^+^ T cells exhibited significantly increased production of IFN-γ and IL-1β (*P < 0.001* for both; **Figures 4F, G**), indicating a more active cytokine effector profile in this cell population. Collectively, these findings indicate that CD8^+^CD68^+^ T cells do not exhibit a simply enhanced cytotoxic phenotype. Instead, they display a composite functional profile characterized by relatively lower expression of cytotoxic molecules, enhanced proliferative capacity, increased tissue residency associated features, and elevated cytokine production. These results further suggest that CD68 expression is closely associated with proliferative, tissue residency associated, and immune effector states in peripheral blood CD8^+^ T cells.

**Figure 4:**
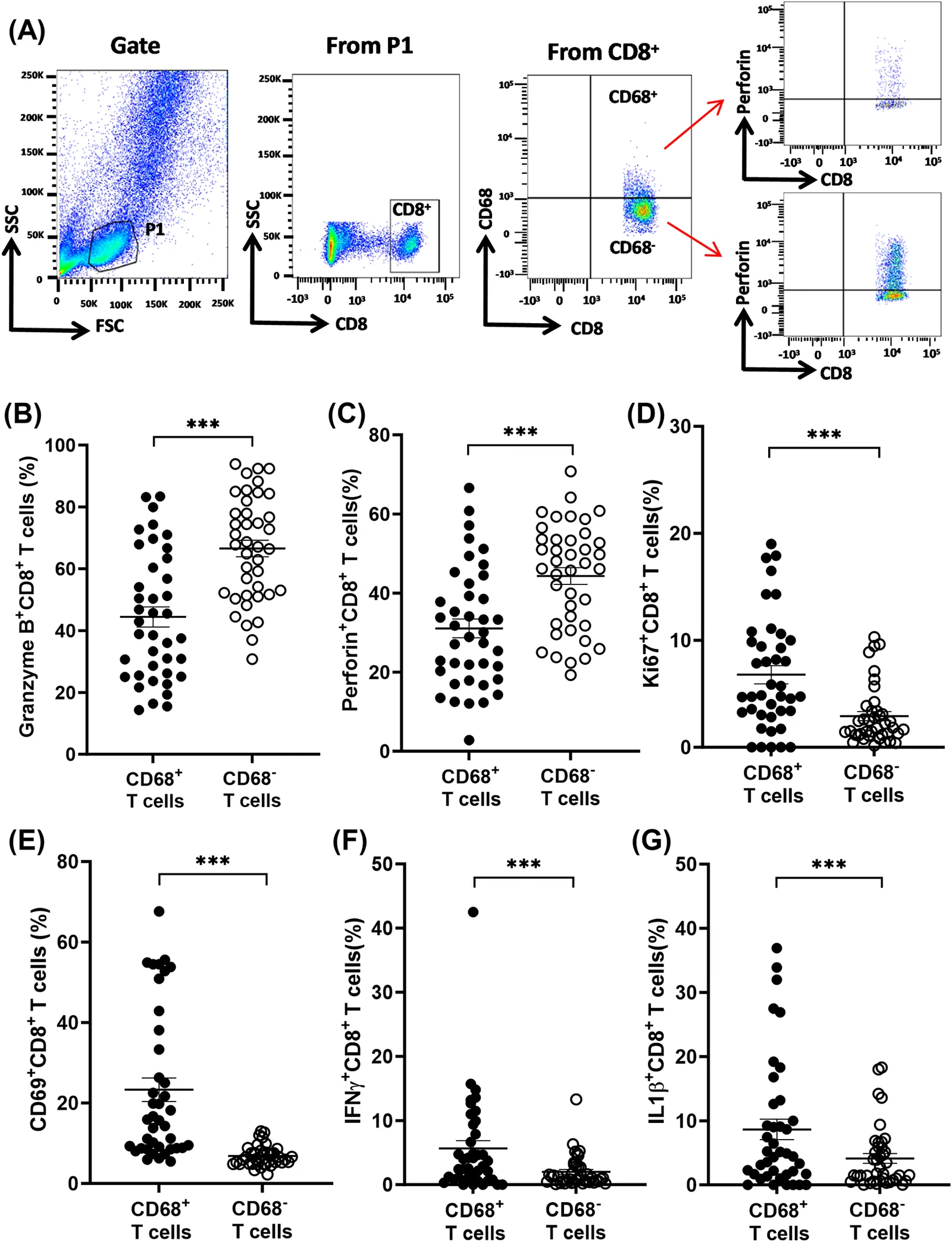
Association of CD68 expression with functional characteristics of peripheral CD8^+^ T cells. **(A)** Flow cytometry gating strategy for analyzing functional molecules in peripheral CD8^+^ T cells based on CD68 expression levels. **(B-G)** Quantitative comparison of expression levels of Granzyme B **(B)**, Perforin **(C)**, Ki67 **(D)**, CD69 **(E)**, IFN-γ **(F)**, and IL-1β **(G)** between CD8^+^CD68^+^ T cells and CD8^+^CD68^-^ T cells. PBMCs used in the experiments were collected from 20 lung cancer patients after completing the first cycle (week 3) of anti-PD-1 therapy. Data are presented as scatter plots showing mean ± SEM. Paired t test, ***P < *0.001*.

## Discussion

Our findings provide preliminary evidence linking lysosome related signaling to T cell states and to the efficacy of anti-PD-1 immunotherapy, while also identifying CD68 as a candidate biomarker with potential clinical relevance. By integrating tumor tissue samples, we first identified gene signatures associated with response to anti-PD-1 immunotherapy based on predefined lysosome related immune and cellular process pathways, and subsequently found that CD68 expression was associated with T cell phenotypes and clinical benefit from anti-PD-1 therapy (**Figure 1**). Consistent with these observations, CD68 expression in peripheral blood CD8^+^ T cells showed a similar association with clinical response (**Figure 2**). This finding is unexpected, as CD68 has been widely reported as a prognostic marker in the tumor microenvironment but studied almost exclusively in macrophages, with its biological significance in T cells remaining largely uncharacterized (10–12). The molecular characteristics of CD68 nevertheless provide a plausible basis for its potential relevance to T cell functional states. CD68 is predominantly localized to lysosomal and endosomal membrane systems, and its expression may reflect lysosomal status (13, 14). Lysosomes and lysosome associated processes, including endocytosis, phagocytosis, autophagy, antigen processing and presentation, and mTOR mediated metabolic regulation, serve as important regulatory hubs for T cell activation, differentiation, and functional adaptation (15, 16). We therefore further examined the distribution and longitudinal changes of CD68 across distinct CD8^+^ T cell subsets (**Figure 3**). CD68 expression displayed subset specific patterns, with higher expression in TN cells than in TEM and TEMRA cells, and was also associated with T cell activation status (**Figure 1F**). Notably, following anti-PD-1 immunotherapy, CD68 expression decreased across multiple CD8^+^ T cell subsets in non-responders, whereas it remained relatively stable in responders (**Figure 3**). These findings raise the possibility that dynamic changes in CD68 may capture alterations in T cell states associated with treatment failure. Collectively, CD68 may represent a dynamic indicator of lysosome related status in T cells, with changes in its expression potentially reflecting the integrated cellular response to persistent antigenic stimulation, metabolic stress, and perturbations in organelle homeostasis. However, the present findings do not establish the presence of lysosomal dysfunction itself, and further mechanistic studies are required to determine whether and how CD68 contributes to or reflects these processes.

Further characterization of CD8^+^CD68^+^ T cells revealed relatively low expression of granzyme B and perforin but higher expression of Ki67 and CD69 (**Figures 4B–E**), suggesting a precursor-like state rather than a terminally differentiated effector phenotype, with greater proliferative potential and capacity for further state transition (17–19). Consistent with previous studies, CD8^+^ T cells with limited cytotoxicity but sustained proliferative and differentiation potential can expand under chronic antigen stimulation and immune checkpoint blockade and give rise to progeny with enhanced effector functions (20, 21). CD8^+^CD68^+^ T cells also exhibited higher IFN-γ and IL-1β expression (**Figures 4F, G**), further suggesting that this population may combine proliferative potential with cytokine producing capacity. Based on these observations, we propose a working model in which a subset of circulating CD8^+^ T cells undergoes activation associated metabolic and lysosome related state remodeling during anti-PD-1 therapy, accompanied by changes in CD68 expression (**Figure 5**). CD8^+^CD68^+^ T cells may represent a precursor-like state characterized by relatively low immediate cytotoxicity, enhanced proliferative potential, and potential tissue-homing capacity. Following appropriate stimulation within the tumor microenvironment, these cells may undergo further state transition and acquire enhanced effector functions, including increased production of granzyme B, perforin, and IFN-γ, thereby contributing to sustained antitumor immunity. They may therefore constitute a circulating reservoir of potentially responsive T cells linked to subsequent tissue associated effector T cell states. This interpretation is consistent with reports that treatment responders tend to have fewer naïve T cells in peripheral blood but more memory T cells in tumor tissues, which is associated with better clinical outcomes (22–25). In particular, circulating cells co-expressing CD69 may possess a greater capacity to respond to tumor-associated signals and contribute to sustained antitumor immunity (26, 27). Overall, our findings provide preliminary evidence that CD8^+^CD68^+^ T cells represent a precursor-like CD8^+^ T cell state with tissue residency associated features. However, this interpretation is currently based primarily on phenotypic and molecular associations, and the lineage relationship, tissue-homing capacity, and functional differentiation of these cells require further validation using lineage tracing and functional studies.

**Figure 5:**
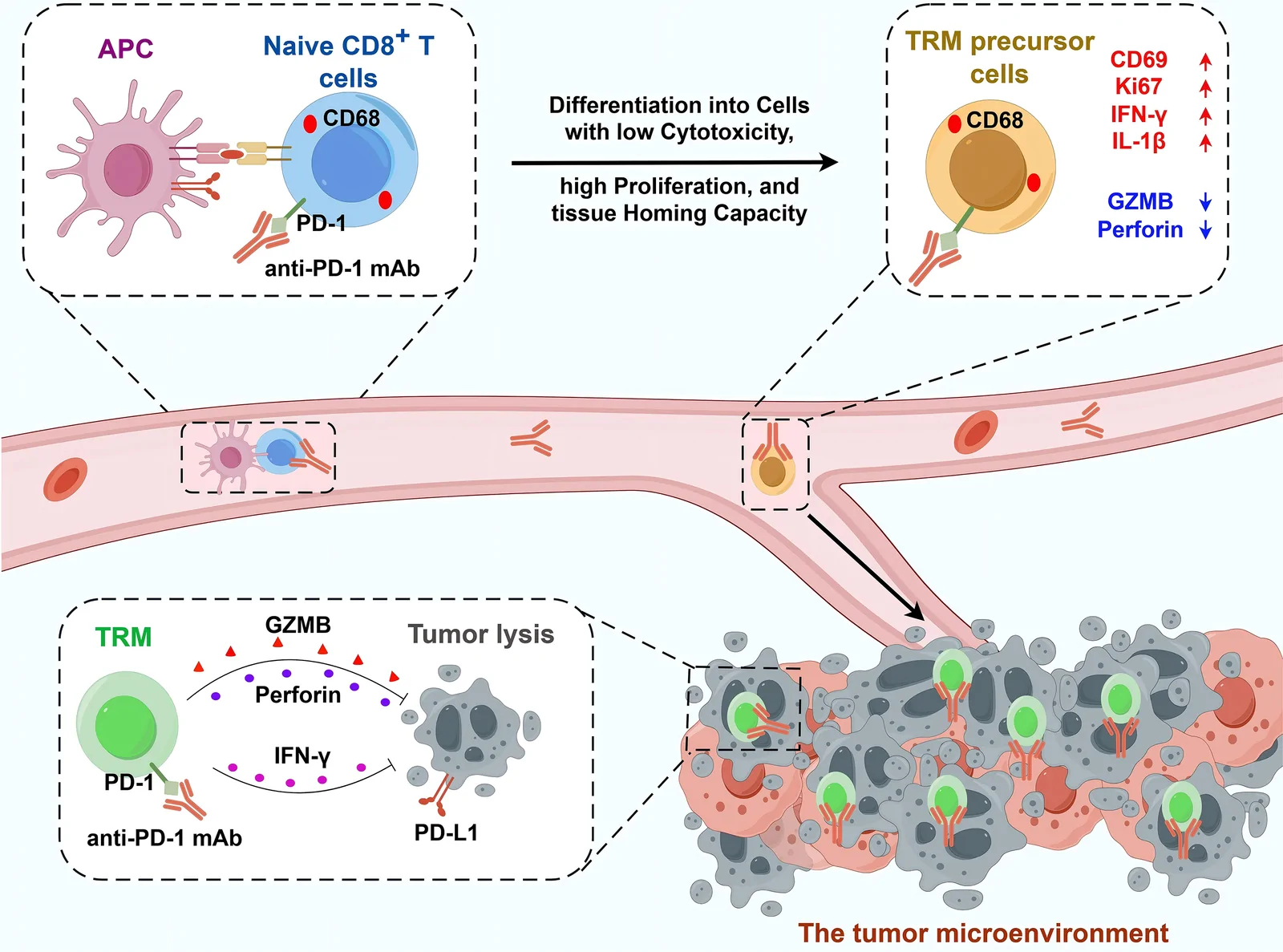
Working model of CD8^+^CD68^+^ T cells as a precursor-like circulating CD8^+^ T cell state with tissue residency associated features during anti-PD-1 therapy. During anti-PD-1 therapy, a subset of circulating CD8^+^ T cells undergoes activation associated metabolic and lysosome related state remodeling, accompanied by changes in CD68 expression. The resulting CD8^+^CD68^+^ T cells may represent a precursor-like state characterized by relatively low immediate cytotoxicity, enhanced proliferative potential, and potential tissue-homing capacity. Following appropriate stimulation within the tumor microenvironment, these cells may undergo further state transition and acquire enhanced effector functions, including increased production of granzyme B, perforin, and IFN-γ, thereby contributing to sustained antitumor immunity. They may therefore constitute a circulating reservoir of potentially responsive T cells linked to subsequent tissue associated effector T cell states. Schematic model created with Figdraw (www.figdraw.com).

From a clinical translational perspective, CD68 can be quantitatively assessed in peripheral blood CD8^+^ T cells, and its longitudinal changes following treatment are associated with response to anti-PD-1 therapy (**Figures 2**, **3**). Compared with repeated tumor biopsies, peripheral blood assessment is minimally invasive, readily accessible, and suitable for longitudinal monitoring, suggesting that CD68 in circulating CD8^+^ T cells may have potential as a liquid biopsy associated immune biomarker for assessing response to immune checkpoint blockade. Moreover, ROC analysis and PFS stratification demonstrated associations between the proportion of CD8^+^CD68^+^ T cells, treatment resistance, and clinical outcomes, providing preliminary clinical evidence for its potential utility. Although these findings remain exploratory, the concordant results from tumor transcriptomic, single-cell, and longitudinal peripheral blood flow cytometric analyses collectively support CD68 as a potential dynamic immune marker. Larger prospective cohorts are warranted to validate its predictive performance and to further clarify the relationship between CD68 associated T cell states and antitumor immune responses.

## Conclusions

We detected a CD8^+^CD68^+^ T cell subset with low expression of granzyme B and perforin and high expression of Ki-67 and CD69, suggesting enhanced proliferative potential and a potential capacity for tissue homing. This phenotype may represent a precursor-like CD8^+^ T cell state with tissue residency associated features. CD68 expression and its dynamic changes in tumor tissues and peripheral blood CD8^+^ T cells were associated with clinical benefit from anti-PD-1 therapy in lung cancer, suggesting that it may serve as a biomarker for reflecting T cell states and monitoring immunotherapy responses. These findings provide a new perspective on response and resistance to PD-1 blockade, although the current evidence is primarily correlational and requires validation in functional studies and larger prospective cohorts.

## Data Availability

The original contributions presented in the study are included in the article/**Supplementary Material**. Further inquiries can be directed to the corresponding authors.
