# Supplementary material for: A CD68-expressing tissue-homing subset in peripheral CD8+ T cells predicts response to anti-PD-1 therapy in lung cancer
Source: Front Immunol. 2026 Oct 13;17:1995008. doi: 10.3389/fimmu.2026.1995008 (PMC13710226; doi:10.3389/fimmu.2026.1995008)
Supplement: Supplementary file 1 [file Table1.docx]

**Supplementary Figure**

**Supplementary Figure 1. PCA score plots of transcriptomic data from three independent cohorts (GSE126044, GSE135222, and GSE166449).** (A) Before batch correction, samples clearly cluster by batch. (B) After batch correction, the batch effect is largely removed. Each dot represents one patient. PC, principal component.

**Supplementary Figure 2. Analysis of the Lysosomal Pathway and Associated Genes in the Validation Cohort**

(A) Volcano plot of differentially expressed genes from the map04142_Lysosome biogenesis pathway in the validation cohort (GSE207422). The eight lysosomal genes screened in the primary cohort are highlighted in red. (B) Heatmap showing the correlation analysis between the aforementioned eight lysosomal genes and the infiltration levels of 10 immune cell types in the tumor microenvironment.
